# P-2118. Pharmacist Led Trimethoprim-Sulfamethoxazole (TMP-SMX) Oral Challenges in a Largely Immunocompromised Population Post Transplant with a Listed Sulfa Allergy

**DOI:** 10.1093/ofid/ofaf695.2282

**Published:** 2026-01-11

**Authors:** Andrea Chin, Kendall J Tucker, Shyam Joshi, YoungYoon Ham

**Affiliations:** Oregon State University/Oregon Health and Science University, Portland, OR; Oregon State University College of Pharmacy, Portland, Oregon; Oregon Health & Science University, Portland, Oregon; Oregon Health & Science University, Portland, Oregon

## Abstract

**Background:**

Medication allergies can limit first-line antibiotic use and sulfonamide allergies are the second most reported medication allergy. Often sulfonamide allergies are not addressed until the patient requires a trimethoprim-sulfamethoxazole (TMP-SMX) course for treatment or prophylaxis. Patients that are immunocompromised, such as solid organ transplant recipients, are at higher risk of developing opportunistic infections such as Pneumocystis jirovecii pneumonia (PJP), toxoplasmosis, and urinary tract infections, where TMP-SMX is the antibiotic of choice. A consideration for allergy challenges in a post-transplant population is that patients may be on immunosuppressant medications potentially compromising the results by masking mild reactions, however, given high clinical need, the lower predictive value is thought to be acceptable.

**Methods:**

Inpatient adults (≥ 18 years) labeled with a sulfonamide allergy who underwent pharmacy-led evaluation and challenged with two doses of TMP-SMX between January 2020 to April 2025 were included. Data collected included the result of the challenge, whether the patient completed a full treatment or prophylactic course of TMP-SMX after a successful challenge, and if the patient was immunosuppressed at the time of the challenge.

**Results:**

Eighty-eight patients received a two-step sulfonamide antibiotic challenge with TMP-SMX. Eighty-one (93%) passed the challenge without an immediate reaction. Sixty-three (85%) subsequently completed a full TMP-SMX treatment or prophylaxis course. Of note one patient reacted during the TMP-SMX challenge yet completed a prophylaxis course with desensitization and symptom management with antihistamines. Four treatment discontinuations were due to delayed reactions (mild maculopapular eruption). Forty-six (75%) of the patients tested had documented immunosuppression use prior to the sulfonamide challenge.

**Conclusion:**

A successful sulfonamide allergy challenge can allow a patient to complete a course of TMP-SMX rather than use a second-line antibiotic. These results demonstrate the safety, efficacy, and benefits of a pharmacist-led antibiotic allergy challenge initiative. Further this can improve health outcomes and implement antimicrobial stewardship for immunocompromised patients.

**Disclosures:**

Kendall J. Tucker, PharmD, MS, Merck: Grant/Research Support Shyam Joshi, MD, Cogent: Advisor/Consultant|Cogent: Honoraria|GSK: Advisor/Consultant|GSK: Honoraria|Guidepoint Consulting: Advisor/Consultant|Guidepoint Consulting: Honoraria|Kalvista: Advisor/Consultant|Kalvista: Honoraria|Nectar Allergy: Advisor/Consultant|Nectar Allergy: Honoraria|Nectar Allergy: Stocks/Bonds (Private Company)|Novartis Corporation Pharmaceuticals: Advisor/Consultant|Novartis Corporation Pharmaceuticals: Honoraria|Takeda: Advisor/Consultant|Takeda: Honoraria

